# A Perspective on the Strategy for Advancing ETVAX^®^, An Anti-ETEC Diarrheal Disease Vaccine, into a Field Efficacy Trial in Gambian Children: Rationale, Challenges, Lessons Learned, and Future Directions

**DOI:** 10.3390/microorganisms12010090

**Published:** 2023-12-31

**Authors:** M. Jahangir Hossain, Ann-Mari Svennerholm, Nils Carlin, Umberto D’Alessandro, Thomas F. Wierzba

**Affiliations:** 1 Medical Research Council Unit The Gambia at the London School of Hygiene and Tropical Medicine, Fajara, Banjul P.O. Box 273, The Gambia; 2 Department of Microbiology and Immunology, Gothenburg University Research Institute (GUVAX), Gothenburg University, 40530 Gothenburg, Sweden; 3 Scandinavian Biopharma, Industrivägen 1, 17148 Solna, Sweden; 4 Section on Infectious Diseases, Department of Internal Medicine, Wake Forest School of Medicine, Winston Salem, NC 27157, USA

**Keywords:** Enterotoxigenic *Escherichia coli*, ETEC, ETVAX, vaccine, efficacy, The Gambia

## Abstract

For the first time in over 20 years, an Enterotoxigenic *Escherichia coli* (ETEC) vaccine candidate, ETVAX^®^, has advanced into a phase 2b field efficacy trial for children 6–18 months of age in a low-income country. ETVAX^®^ is an inactivated whole cell vaccine that has gone through a series of clinical trials to provide a rationale for the design elements of the Phase 2b trial. This trial is now underway in The Gambia and will be a precursor to an upcoming pivotal phase 3 trial. To reach this point, numerous findings were brought together to define factors such as safe and immunogenic doses for children, and the possible benefit of a mucosal adjuvant, double mutant labile toxin (dmLT). Considering the promising but still underexplored potential of inactivated whole cells in oral vaccination, we present a perspective compiling key observations from past ETVAX^®^ trials that informed The Gambian trial design. This report will update the trial’s status and explore future directions for ETEC vaccine trials. Our aim is to provide not only an update on the most advanced ETEC vaccine candidate but also to offer insights beneficial for the development of other much-needed oral whole-cell vaccines against enteric and other pathogens.

## 1. Introduction

Currently, there are World Health Organization (WHO) prequalified inactivated whole cell vaccines against cholera, an enteric pathogen (i.e., Euvichol, EuBiologics Co., Ltd., Sinsa-dong, Gangnam-gu, Republic of Korea; Shanchol Sanofi Healthcare India Private Limited, Telangana, India; Dukoral, Valneva Sweden AB, Solna, Sweden). Orally administered vaccines using inactivated bacterial cells offer an opportunity to realize the application of this approach to other enteric pathogens, like ETEC and *Shigella*, which has yielded encouraging results [1,2,3,4,5,6,7]. These results are helping to develop further standalone and combination ETEC and *Shigella*-containing vaccines [5,8]. For ETEC, this approach is now being applied to bring the most advanced ETEC vaccine, ETVAX^®^, into a Phase 2b trial in Gambian children. For ETVAX^®^ or other inactivated whole cell vaccines to be tested successfully in the often difficult-to-immunize target populations of young children in low- and middle-income countries (LMIC) requires the resolution of several challenges to ensure safety together with an adequate immune response to protective antigens.

This manuscript will describe how a series of clinical trials with ETVAX^®^ have been used to work out a strategy to advance ETVAX^®^ into the first field efficacy trial for an ETEC vaccine in the last two decades. The challenges addressed in this development path and lessons learned will serve as an important roadmap in guiding further evaluation of ETVAX^®^ in anticipated pivotal Phase 3 trials for licensure. In addition, this perspective will highlight several potentially important observations made in past and more recent trials of ETVAX or its precursors that have implications on the design and the range of research questions to be addressed by the upcoming Phase 3 trials of ETVAX. This includes additional knowledge to be gained on the vaccine induced ETEC immunity and its impact on protection against infection and disease. It will also be a guide for other ETEC and enteric vaccine developers as they attempt to move their candidates into late-stage clinical trials.

In the 1990s, a series of age-descending safety trials of a first-generation ETEC vaccine was conducted in Egyptian adults and children, followed by an efficacy trial in Egyptian children [9,10,11]. This first-generation vaccine was an orally administered, inactivated, multivalent whole-cell vaccine that included ETEC strains expressing colonization factors (CFs) CFA/I, CS1, CS2+CS3, CS4, CS5, along with a recombinant toxin component, rCTB. CTB is the non-pathogenic B-subunit of Cholera Toxin (CT). CT is similar in structure and function to heat-labile enterotoxin (LT).

The efficacy trial conducted in Egyptian children was randomized, double blind, and placebo-controlled [12]. Children aged 6–18 months received 3 administrations of the vaccine or placebo at two-week intervals. They were then monitored for diarrhea by twice-weekly home visits for one year. The primary endpoint was the time to the first diarrhea episode associated with ETEC producing LT and/or heat-stable enterotoxin (ST), along with a vaccine-shared colonization factor. Among the 314 children who received three doses of the vaccine (152) or placebo (162), 31 and 40 ETEC infections with mostly mild diarrhea were observed in the vaccine and control groups, respectively. The adjusted vaccine efficacy was 20% (95% CI: −29% to 50%), suggesting the vaccine did not provide significant protection against vaccine-homologous ETEC diarrhea. Nevertheless, the same vaccine was found to be safe, immunogenic, and afforded 77% protection against severe ETEC disease outcomes in US adults travelling to Mexico and Guatemala [13]. A follow-on study of a new formulation of this vaccine, with an rCTB component produced from a new construct also showed protective efficacy against more clinically significant ETEC Traveler’s Diarrhea (TD) in another cohort of travelers to Mexico and Guatemala [14]. However, since the vaccine was not effective in Egyptian children, the development of this first-generation vaccine was discontinued.

The WHO organized meetings in 1998 and 2003 to review the epidemiology and progress toward ETEC vaccine development [7]. Referring to the first-generation whole-cell vaccine, the WHO stated that “further studies of the killed oral ETEC vaccine are warranted in the target population aged under 12–24 months, given the good safety and immunity record.” More specifically, it was recommended that exploration should be made into “a suitable adjuvant or delivery form and/or increasing the amounts of protective antigens, particularly CFs, on the bacterial surface.” Moreover, it was recommended that endpoints against severe disease or hospitalization associated with vaccine-specific antigens be considered [7].

## 2. Strategy Development for Use of ETVAX^®^ in Children in LMIC

ETVAX^®^ is an inactivated whole-cell vaccine with genetically modified *E. coli* strains that overexpress CFA/I, CS3, CS5, and CS6 [15]. Globally, these CFs are the most common, and this vaccine could be used in many low- and middle-income countries [16,17,18,19]. The vaccine also includes a hybrid LCTBA toxoid, which is a hybrid of the B-subunit of LT and the B-subunit of CT [20]. A double amino acid mutant of LT, dmLT, was also added to the vaccine as a mucosal adjuvant [21] to enhance antitoxin and anti-CF immunity. Importantly, this improved vaccine formulation could provide cover for 70% to 80% of the ETEC strains associated with morbidity and mortality in LMICs, as well as with international travelers and deploying military personnel [2]. In addition, ETVAX addresses all the WHO recommendations for an improved ETEC vaccine formulation of increased colonization factor amount per dose, the inclusion of immunogenic CS6 and a mucosal adjuvant in the formulation [7]. The vaccine has been tested among Swedish adults and Bangladeshi and Zambian adults and children, as well as in Finnish adults, and was found to be safe and immunogenic [22,23,24,25] (Table 1).

## 3. Efficacy Trials

As previously noted, the first-generation vaccine was not efficacious in children. However, there are indications that the trial design, rather than the vaccine’s content and formulation, may have contributed to the disappointing results. As children were identified early in the course of illness by twice-weekly home visits and thus treated promptly, cases did not progress to more severe illness. All cases were mild. The first-generation ETEC vaccine may have been effective against moderate to severe ETEC diarrhea as was demonstrated by early rotavirus vaccine trials that showed greater protection against more severe than less severe disease [30]. Similarly, earlier formulations of the current ETVAX were also more effective against more clinically significant ETEC-associated illness in travelers [13,14].

Given the positive safety record of ETVAX^®^, a phase 2b efficacy trial is currently underway in The Gambia. This trial is expected to provide evidence of safety, support building a large safety data base for eventual licensure, expand knowledge of immunogenicity, and offer preliminary evidence of efficacy as groundwork for a pivotal phase 3 efficacy trial. The Gambian trial is double blind, randomized, and placebo-controlled study and involves just under 5000 Gambian children aged 6 to 18 months. Children received three oral ETVAX^®^ doses or placebo on study days 1, 15, and 90. Depending on the date of enrollment, they are followed for 12 to 24 months. Acute diarrhea is defined as four or more loose or liquid stools in 24 h with an onset within the previous 10 days and after three days without diarrhea. ETEC primary endpoints are children having moderate-to-severe dehydrating diarrhea with ETEC expressing vaccine homologous antigens (LT- or LT/ST-enterotoxin, or ST with at least one CF (CFA/I, CS3, CS5, CS6)) without culture-confirmed *Shigella*, PCR-confirmed rotavirus, norovirus GII, and cryptosporidium co-pathogens. For the primary endpoint, diarrhea cases are counted from seven days after the third vaccine dose until study end. In contrast to the first-generation efficacy trial, diarrhea surveillance is passive, with mothers bringing children to one of seven study clinics for evaluation and treatment.

## 4. ETVAX^®^ Study Design

Below, we describe the reasoning for several key trial design issues important to the trial in The Gambia. These issues include study location, vaccination age, vaccine dose and number of doses, definition of vaccine-preventable outcomes, and provide an update on the status of the ETVAX^®^ clinical trial. We anticipate that this information will be instrumental in understanding this trial design and useful for designing future field-based efficacy trials for enteric vaccines.

## 5. Trial Site Selection

The Gambia was selected as the trial site and The Medical Research Council Unit The Gambia (MRCG) at the London School of Hygiene & Tropical Medicine (LSHTM) is the implementing organization.

The Gambia is a small West African country covering an area of about 11,000 square kilometers, with a population of about 2.5 million people [31]. The median age is 17 years [32]. Life expectancy at birth is 62 years [33] and infant mortality rate is 34/1000 live births [34]. The mean per capita income of The Gambia is US $772 per annum [35], below the sub-Saharan Africa average of US$ 1633 [36] and 17.2% population live in poverty [35].

The Gambia has a documented high burden of ETEC diarrhea [37,38,39]. In a study partly conducted in The Gambia, 4.9%, 8.0%, and 9.2% of 400, 455, and 174 children aged 0–11, 12–23, and 24–59 months respectively had moderate-to-severe diarrhea attributable to ETEC expressing STh or LT/ST (cases with STp were not included). The weighted annual incidence for the same age groups were 0.7, 1.5, and 0.3 per 100 child-years [39]. Results from a study exploring the etiology of acute and persistent watery diarrhea following introduction of rotavirus vaccine in three sub-Saharan countries, including The Gambia, mirrored these results [37].

Over the past 75 years, MRCG has developed and maintained programs, infrastructure, and resources that support a wide range of studies, from basic science to the evaluation of interventions for the control of diseases of public health importance in sub-Saharan Africa. The Unit’s investigator-led research is underpinned by the combination of excellent laboratory facilities, access to well-defined populations supportive of MRCG research, excellent clinical services, rigorous ethical procedures, and the ability to deliver Good Clinical Practice (GCP)-compliant clinical trials. Previous field vaccine trials include, but are not limited to, an efficacy trial of nine-valent pneumococcal conjugate vaccine trial [40,41], a trial of a Meningococcal ACWYX Conjugate Vaccine [42], and a trial of PCV-7 on pneumococcal carriage [43].

The laboratory facilities at MRCG serve as a center of excellence for training in laboratory science within the West African region. Two MRCG laboratories support this trial, including a field laboratory in Keneba, one of the two MRCG field stations across The Gambia, and the central MRCG laboratory in Fajara located in the Lower River Region and West Coast Region, respectively. These laboratories have the capacity for microbiology, immunology, and the Fajara Laboratory for serology and molecular diagnostics as well. Both laboratories hold Good Clinical Laboratory Practices (GCLP) accreditation (Qualogy GCLP Accreditation, Kettering, UK); generate high-quality data using standardized operating procedures, quality control and quality assessment procedures. They have also successfully passed external monitoring assessments. The Fajara and Keneba facilities can store samples at −80 degrees Celsius and have the expertise in packaging and shipping stool samples for Finland and Sweden for further testing and quality control.

## 6. Vaccine Dose and Number of Doses Administered

To select a vaccine dose (i.e., number of inactivated bacteria) and the number of doses for The Gambia efficacy trial, investigators reviewed the safety and immunogenicity results from two randomized, placebo-controlled ETVAX^®^ trials conducted in low-income countries. The first trial was conducted in Dhaka, Bangladesh [23], and the second in Lusaka, Zambia [24]. Both trials used a dose-escalation, age descending design.

Bangladesh: Healthy children were divided into three age groups: 24–59 months (n = 130), 12–23 months (n = 100), or 6–11 months (n = 200) [23]. Children in each group received two doses, two weeks apart, of a placebo or a vaccine. Vaccine was administered at one of the following three doses: approximately one-half an adult dose [5.5 × 10^10^ bacteria + 500 µg LCTBA], one-quarter of an adult dose [2.5 × 10^10^ inactivated *E. coli* bacteria +250 µg LCTBA], or, for the youngest age group, one-eighth of an adult dose [1.25 × 10^10^ bacteria + 125 µg LCTBA]. The vaccine was given without or with dmLT adjuvant at doses of 2·5 μg, 5.0 μg, or 10.0 μg. Vaccine was suspended in bicarbonate buffer in volumes of 30 mL, 15 mL, and 10 mL for children 24–59, 12–23, and 6–11 months, respectively at 1× strength. Placebo recipients received the same volume as vaccine recipients.

When correlating dose to immunogenicity, the analysis of mucosal immune responses was insightful. Specifically, the IgA antibodies in lymphocyte secretions (ALS) were measured in two age groups: children aged 24–59 months and those aged 12–23 months. The results showed that two vaccinations with ETVAX^®^ alone, either a quarter or half dose, or half doses with different doses of dmLT, elicited high and significant IgA ALS responses. These responses were against all four vaccine colonization factors and LTB.

The ALS IgA responses against the vaccine antigens in infants aged 6–11 months were lower than in older children but significantly higher in vaccine recipients than placebo recipients for CFA/I, CS3, and LTB. However, significant secretory IgA (sIgA) responses in feces, as a more direct measure of intestinal immune responses, were induced against all five vaccine antigens in the infants; these responses did not significantly differ with a one- quarter and one-eight vaccine dose.

Most solicited adverse events (AE) were mild and not greater than moderate in the week after either dose. Vomiting was the most common AE and its frequency decreased with increasing age (17% in those aged 6–11 months, 13% in those aged 12–23 months, and 8% in those aged 24–59 months after the highest selected dose, i.e., half dose for the older and a quarter dose for the infants). A lower vaccine dose was associated with a reduced risk of vomiting (i.e., vomiting in children aged 6 to 11 months: 33% with a half dose, 17% with a quarter dose, and 7% with an eighth dose). Adding dmLT did not modify the safety profile.

Zambia: Children were divided into two age groups: 10–23 months (n = 60) and 6–9 months old infants (n = 146). They received three doses of either placebo or vaccine on study days 1, 15, and 90, instead of two doses [24]. An additional dose, as compared to the Bangladeshi trial, was added at day 90 as previous studies suggested that oral vaccination with inactivated bacteria is most effective when multiple doses are given [44] and that two doses of ETVAX induced significant immunological memory against all vaccine antigens to a third dose in Swedish adults [45]. Vaccine doses in both age groups were either one-quarter or one-eighth of an adult dose, each administered together with 2.5 μg dmLT and in 10mL bicarbonate buffer, 2× buffer strength.

For immunogenicity, limited to analyses of plasma, the data showed that for older children, there was no statistically significant difference between vaccine and placebo recipients in terms of plasma IgA response from baseline to after vaccination, (i.e., ≥two-fold increase) regardless of the dose for any of the vaccine colonization factors (CFs). However, both IgA and IgG responses against LTB were significantly higher in vaccine than in placebo recipients.

In infants, the frequencies and magnitudes of plasma IgA responses from baseline were statistically greater in vaccine recipients than placebo recipients for the one-fourth dose against CFA/I, CS3, CS5, and LTB, and for the one-eighth dose against CS3 and LTB. Among the infants, a third dose (one-fourth of an adult dose) induced a significant increase in the magnitudes of plasma IgA antibody response against LTB, CFA/I, CS3, and CS5, whereas the second dose did not.

The lack of significant immune responses in the vaccine recipients against most of the CFs in older children might be explained by the unusually high frequencies of immune responses against ETEC antigens in the placebo group, possibly reflecting a high rate of asymptomatic ETEC infections in this age group.

The vaccine was safe in Zambian children. The majority of solicited adverse events were mild or in a few instances moderate in the seven days after any dose, and no statistically significant differences were observed in the frequencies of vomiting, fever, or diarrhea between vaccine and placebo recipients regardless of age, dose, or number of doses.

Application of dosing data to Phase 2b field efficacy trial in Gambia: Findings from the Bangladesh trial revealed significant outcomes. A quarter dose of the vaccine in older children and infants induced notable increases in mucosal immune responses against LTB and the vaccine CFs. Additionally, in Zambian infants, a quarter dose led to statistically significant increases in plasma immune responses. These responses were further boosted by a third dose. A quarter dose in Bangladeshi infants was associated with reduced vomiting compared to a half dose, and Zambian infants showed nonsignificant rates of vomiting after a quarter and eighth doses when compared to placebos. These findings suggest that a quarter dose was well-tolerated and immunogenic in Bangladeshi and Zambian children. In conclusion, for the Gambia trial, the highest dose selected was one-quarter of an adult dose with 2.5 μg of dmLT, administered in three separate doses. Multiple vaccine doses increase the probability of a vaccine response, and the timing between doses can be adapted to fit with routine vaccination schedules.

## 7. Use of an Adjuvant

The detoxified LT molecule is not only a potent and antigenic toxin component for a future ETEC vaccine that may contribute significantly to improved protective vaccine efficacy, reduced disease severity and broader protection [46,47,48], but it is also an effective mucosal adjuvant [21,49]. Since young children in LMICs respond less well compared to older children with oral bacterial vaccines, it was important to evaluate the safety and utility in vaccinating children with ETVAX^®^. Adjuvant data from 6 to 11-month-old Bangladeshi infants showed that dmLT improves the frequency and the magnitude of the mucosal immune response to ETEC antigens following immunization with the killed whole cell vaccine, ETVAX^®^. Furthermore, in Bangladeshi infants, the kinetics of the mucosal immune response were accelerated by the inclusion of dmLT in the vaccine. These studies in Bangladeshi infants have also shown that the dmLT adjuvant can improve the mucosal antibody response to both protein (CS6) and polysaccharide antigens (O78 lipopolysaccharide) [29,50]. Further, the use of dmLT was particularly important for ETEC vaccine “take” in 6 to 11-month-old infants, as fractional doses of the vaccine needed to be used to improve its tolerability [30]. In more in-depth immunological studies, the inclusion of dmLT in the vaccine formulation improved expression of T cell responses to key ETEC antigens [51].

## 8. Age at Vaccination

Since ETEC is one of the first enteric infections experienced by children in LMICs and considering the need for protection through early life when ETEC incidence is highest, initiating vaccination against ETEC as soon as possible after birth is crucial [52]. However, when it comes to vaccinating infants, several factors can influence how early a vaccine can be administered. These factors include knowledge of local ETEC incidence by age, the vaccine’s immunogenicity, safety, and reactogenicity profile in very young children, and host conditions that may inhibit immune responses, such as the presence of comorbidities, malnutrition, and maternal antibodies [53,54].

As for the age of administration, ETVAX^®^ can be given to children as young as six months old, as demonstrated by trials conducted in Bangladesh and Zambia [23,24]. The vaccine was rarely reactogenic, and no safety concerns were observed in infants and older children. Data from the ongoing Gambia trial also suggests that ETVAX^®^ can be considered safe and non-reactogenic in children and infants (see trial update in this paper). Given this safety record, future studies could probably investigate the immunogenicity, safety, and protective efficacy of ETVAX^®^ in children younger than six months. This could potentially allow for its integration into childhood vaccination schedules.

In the Gambian trial, the first dose of ETVAX^®^ is administered to children aged between 6 and 18 months. Depending on their enrollment date, children are followed up for a period of 12 to 24 months. Therefore, children are monitored for diarrhea from the age of six months up to nearly 42 months. This period covers the peak ages for ETEC infections in Gambian children [40], thereby allowing for the estimation of vaccine efficacy by age.

When an infant is vaccinated, maternal antibodies transferred from the mother to the child, either transplacentally or through breast milk, may inhibit the host’s response to the vaccine. It has been demonstrated that the presence of transplacental antibodies impede immune responses to some vaccines used in routine immunization programs [54,55,56]. While the impact is less clear, antibodies from breastfeeding may also inhibit vaccine responses [56,57]. The levels of transplacental antibodies gradually decline overtime [58], and antibodies from breast milk wane or disappear when breastfeeding is discontinued. The broad age range for vaccination in this trial offers an opportunity to observe age-specific efficacy among children vaccinated as infants when maternal antibodies are present, and those vaccinated at older ages when these antibodies have likely waned.

In summary, giving the first ETVAX^®^ dose at six months with coverage up to 18 months should allow sufficient time to evaluate vaccine efficacy during the period of highest incidence.

## 9. Vaccine Preventable Endpoints and Cross Reactivity

In a clinical trial, a clinical endpoint is a targeted outcome that is detected and analyzed to determine the efficacy or safety of the product [59]. In the trial in The Gambia, vaccine safety is one of primary endpoints while the vaccine-preventable endpoint (VPO) for efficacy is moderate-to-severe diarrhea in young children associated with ETEC bacteria expressing antigens homologous with vaccine CFs and toxin components. The endpoint was limited to moderate and severe cases as the vaccine is thought to primarily prevent disease severity in infected children, although it cannot be excluded that in some cases the vaccine prevents infection. Prevention of infection was observed in travelers given a precursor of the ETVAX vaccine, but this impact has not been evaluated in children [14]. More specifically, the primary endpoint is the prevention of moderate-to-severe diarrhea associated with ETEC strains expressing LT- or LT/ST, or ST enterotoxin with at least one CF (i.e., CFA/I, CS3, CS5, CS6). For example, a common infecting vaccine preventable endpoint would be an infection with ST-CS6. As ETEC strains may express more than one CF, including a CF antigen not added to the vaccine such as CS1 (for example, expressing CS1 + CS3), it is further anticipated that ETVAX^®^ endpoints will include these strains despite the vaccine containing only one of vaccine antigen (i.e., CS3).

Secondary endpoints provide further information on the effectiveness and safety of the vaccine. A secondary efficacy endpoint is protection against moderate-to-severe diarrhea caused by any ETEC strains, as ETVAX^®^ is expected to have broader protection as some vaccine CFs share conserved regions that have been identified through genetic and phylogenic studies [18,23,60]. These regions induce cross-reactive antibodies that recognize multiple CFs. For example, shared cross-reactive epitopes have been identified within the CFA/I-like family, including CFA/I, CS1, CS2, CS4, CS14, and CS17 [61,62], and the CS5-like family, including CS5, CS7, CS18, and CS20 [18,60,63]. As there is potential for cross-reactive antibodies, this would increase the potential coverage of ETVAX^®^. It has been suggested that cross-reactive CF antibodies could increase coverage up to 70% of all CF positive ETEC, and by including antibodies against LT, up to 85% of all clinical ETEC isolates [18]. Importantly, antibodies cross-reactive with other CS antigens related to the CFA/I component in the vaccine have been found in Swedish adults and in Bangladeshi and Zambian children after immunization with ETVAX [30]. We would anticipate a similar broader CF/CS response in Gambian infants vaccinated with ETVAX and it will be important to determine if broader disease protection is also observed, since this trial provided the first opportunity to address this question in LMIC infants and children.

## 10. Trial Update

This trial was implemented in the North Bank, Central River, and Lower River Regions of The Gambia, recruiting 4936 children aged 6-18 months over a period of 16 months. Passive surveillance for acute moderate-to-severe diarrhea ended in October 2023. For safety monitoring, 350 children were actively visited at their homes within seven days of receiving each of the three doses. Active surveillance identified 88 adverse events, two of which were severe and considered to be related to the product (one case of vomiting and one of fever). Among all other children (n = 4586), there were 420 adverse events; four of them were severe but not considered related to the product. There were 47 serious adverse events primarily due to respiratory illnesses (47%) and diarrhea/gastroenteritis (19%). However, none of them was deemed product-related. As of 20 June 2023, 604 cases of moderate-to-severe diarrhea have been detected. Testing for ETEC phenotypes and co-pathogens is ongoing. Although the data remain blinded, ETVAX^®^ appears safe, with most adverse events classified as mild or moderate. Efficacy results will be available in 2024.

## 11. Future Directions

For the first time in over two decades, a vaccine candidate targeting ETEC has advanced to an efficacy trial for young children in a low-income country. The results and insights from this trial have the potential to inform the design of field trials against diarrhea-causing pathogens in low- and middle-income countries. Here, we explore several future directions for ETEC and other diarrhea vaccine trials.

## 12. ETEC Detection and Developing ETEC-Specific Clinical Severity Measures

The identification and characterization of ETEC in diarrhea stool samples traditionally rely on culturing and analyzing E. coli strains. Phenotypic approaches, such as GM1-ELISA for toxin detection and monoclonal dot-blot assays for colonization factors (CFs), are commonly employed and supplemented with molecular techniques like Polymerase Chain Reaction (PCR) [64,65,66]. There is an increasing interest in determining whether molecular methods can completely replace traditional culture, ELISA, and dot-blot assays in diagnosing and characterizing strains.

Evidence from an ETVAX trial in travelers to Benin, West Africa [25], illustrates the potential of molecular methods in this context. A study using a real-time PCR-based TaqMan Array Card (TAC) detected more ETEC cases in culture-negative stool samples (138 out of 577 negatives) than traditional culture methods. It also demonstrated a strong correlation with ELISA when identifying toxin types (achieving 83% agreement in 168 out of 203 samples). Furthermore, TAC exhibited high performance in CF detection when compared to dot-blot results, showing 98% sensitivity and 92% specificity across 1025 samples [67]. These findings suggest that the TAC can effectively be used to detect and characterize ETEC strains, potentially offering a more efficient and sensitive alternative to conventional methods.

Beyond the identification of ETEC, when assessing the efficacy of an oral vaccine against ETEC-associated diarrhea, it is crucial to evaluate the clinical severity of the episode not simply the presence of the enteropathogens, as an oral vaccine is likely more effective against moderate to severe diarrhea than mild diarrhea. Therefore, the capacity of research staff to accurately evaluate the degree of diarrhea severity (e.g., mild, moderate, severe) is vital. Several quantifiable scoring systems have been proposed and evaluated to measure clinical severity, including, but not limited to, the World Health Organization [68], Vesikari [69], CODA [70], Clark [71], Dhaka [70], and CIDRZ [72] systems. These scoring systems largely rely on clinical evaluation and mathematical scoring of selected signs and symptoms. For rotavirus diarrhea trials, the Vesikari score has been widely adopted [69,73]. However, this severity score may not be suitable for ETEC or other bacterial enteropathogens (e.g., *Shigella*) as it is less likely to induce vomiting, which is a key feature of rotavirus infection and the Vesikari score. Despite the availability of numerous scoring systems, there is no clinically validated score specific to ETEC-associated diarrhea in children, although an ETEC-specific clinical severity score has been proposed for ETEC vaccine trials in adult travelers, based on encouraging results after initial application [14] that may provide some guidance to score development. While it may be possible to use a scoring system as is or to adapt a scoring system appropriate for acute watery diarrhea, it is advisable that future ETEC-specific clinical scores are developed and validated to minimize potential misclassification of ETEC disease severity.

## 13. Measuring Immune Markers

While field trials like the Gambia study are essential to estimating vaccine efficacy and obtaining licensure or prequalification, identifying immune markers such as serum antibody titers that predict or correlate with vaccine efficacy can simplify and expedite vaccine development. Use of these markers reduce sample size requirements, provide a standardized approach to evaluating vaccine protection across diverse trial populations, and present insight into the immune response generated by the vaccine. There are few studies documenting ETEC immune markers from natural infections or vaccine trials. However, epidemiological studies of natural infection have demonstrated a relationship between titers against ETEC antigens and incidence of ETEC diarrhea [74]. A case-control study in Egyptian children, measuring IgG serum titers against CFA/I showed a reciprocal IgG serum titer greater than or equal to 76 was associated with a 77% reduction in odds of CFA/I ETEC-associated diarrhea [75]. An ETEC efficacy study of adult travelers from the United States to Guatemala and Mexico revealed a link between anti-CTB IgA titers measured upon arrival and the risk of moderate to severe ETEC diarrhea (Table 2). In this trial, anti-CTB markers showed an inverse linear trend between the relative risk of ETEC diarrhea and reciprocal CTB titers such that efficacy reaches 84% (i.e., 1.0–0.16×100) or more for anti-CTB titers greater than 361. It was also interesting to note in this trial that higher level of anti-CTB serum IgA antibodies were also associated with a reduced risk of infection with ETEC strains matching with the vaccine, as well as with *Campylobacter* [14]. Higher levels of anti-CTB IgA antibodies were also associated with broader vaccine protection against all-cause diarrhea [14]. Data suggest that ETEC antigen-specific antibody titers correlate with protection against both ETEC and possibly other pathogens to some extent, but additional population-based pediatric studies and vaccine trials in endemic ETEC populations are needed to validate immune markers for use in vaccine studies. Such studies could be incorporated into ETEC trials.

## 14. Conducting Proteomic Microarrays

Proteomic microarrays are high-throughput tools in biological and clinical research that are used to study the interaction and activities of proteins and to determine their function on a large scale [77]. These microarrays can potentially analyze tens of thousands of proteins in a single experiment. These tools are particularly useful in vaccine research, allowing for the identification of antigen targets, analysis of immune responses to protein antigens, comparison of antibody responses between different vaccine candidates, observation of changes in antibody expression pre- and post-vaccination, discovery of markers for vaccine efficacy, and modification of vaccine formulations based on responses. Specifically, in ETEC vaccine research, microarrays facilitate the investigation of antibody responses to a wide range of ETEC protein antigens. This enables researchers to assess the breadth and depth of vaccine responses, identify antigens correlated with vaccine immunogenicity, protection, and safety.

In a phase 1 randomized controlled trial of ETVAX, IgG antibody responses to over 4000 ETEC antigens and surface proteins were evaluated in twenty Zambian children, aged 10 to 23 months, including four from the placebo group [78]. The study compared immune responses before and after vaccination. It was observed that post-vaccination reactivity to CFA/I, CS3, CS6, and LTB (vaccine-associated antigens) was significantly stronger than baseline in the vaccinated group compared to the placebo group. Three non-vaccine CFs (i.e., CS4, CS14, and PCF071) were among the most reactive antigens in the vaccinated group, suggesting cross-reactivity and the potential to expand the breadth of ETVAX coverage, corroborating previous studies [18].

Furthermore, a challenge-rechallenge experimental human infection trial used an ETEC challenge strain (H1047) and a proteomic array with 957 antigens. This trial detected serum and mucosal responses suggesting protection not only against common vaccine antigens (CFA/I, LTB) but also against antigens not typically targeted in ETEC vaccines, such as EtpA, EatA, YghJ, flagellin, and a pertactin-like autotransporter [79].

These findings indicate that future studies utilizing proteomic microarrays could be instrumental in advancing the understanding of ETVAX.

## 15. Measuring Protection against Other Diarrhea Pathogens

There are indications that ETVAX could have broader protection than against ETEC-associated diarrhea alone, and efficacy studies could consider secondary endpoints other than ETEC or, for example, ETEC- or *Salmonella*-associated diarrhea. As mentioned above, an efficacy trial among US adults visiting Mexico or Guatemala using the first generation ETEC vaccine, suggested that travelers were at reduced risk of *Campylobacter* and *Salmonella* diarrhea when reciprocal anti-CTB serum IgA titers were greater than 360 [76]. The reduction in risk was not seen with ETEC strains that were not homologous with vaccine antigens suggesting the reduction was specific to these other infections. In the same study, among vaccine responders, those with severe diarrhea (severity score of 5 or 6) regardless of cause, protection of greater than 48% was observed. Other studies have shown that vaccination with CTB containing vaccines may affect illness due to *Campylobacter* and *Salmonella* [14,80].

## 16. Evaluating Vaccine Herd Protection

A deeper understanding of how to use vaccines can help to protect the community as well as the individual [81]. To this end, ETEC field trials may consider measuring not only the direct protection of the vaccine on vaccinated individuals, but also the protection afforded to the unimmunized due to herd protection (also known as herd immunity). Herd protection arises when a significant percentage of a population achieves a threshold of immunity through vaccination or exposure thereby reducing opportunities for transmission and offering some protection to the unexposed and unimmunized [81,82]. As vaccine products progress to field efficacy or post-licensure studies, investigators can incorporate study methods that measure herd protection such as cluster-randomized trials [83]. Rotavirus, typhoid, and cholera vaccine studies have used a diversity of approaches in field trials for measurement of herd protection [84,85,86,87,88,89,90].

## 17. The Gut Microbiome and Vaccine Efficacy

The burgeoning field of research into the interaction between oral vaccines and the gut microbiome is revealing significant insights. Studies increasingly suggest that gut microbiota composition can enhance the efficacy of vaccines. Additionally, there is evidence that oral vaccines may influence the gut microbiome.

In the case of oral rotavirus vaccines, research conducted in infants from diverse geographical regions such as Ghana, Pakistan, and Nicaragua indicate that the gut microbiota’s composition could predict the vaccine’s effectiveness [91,92,93]. Likewise, studies on oral cholera vaccines have linked the gut microbiome’s composition at the time of vaccination with the development of memory B cells, vital for sustained immunity [94]. This underlines the potential critical role of the microbiome in modulating the immune response to vaccines.

Moreover, interventions targeting the gut microbiota, such as the administration of prebiotics and probiotics, have been shown to enhance the immune response to oral cholera vaccines. For instance, in a humanized mouse model mimicking child undernutrition, these interventions improved responses to cholera vaccines [95]. Additionally, the first-generation ETEC vaccine in adult travelers has demonstrated protection not only against ETEC-associated diarrhea but also against infections like *Campylobacter* and *Salmonella* [14]. This might be attributable to non-specific immune responses to these bacteria or a broader impact on the gut microbiome that reduces susceptibility.

These developments hold significant implications for ETVAX. Given the evident role of the gut microbiota in vaccine response, similar interactions might be relevant for an oral ETEC vaccine. Understanding these interactions could be key to enhancing its efficacy, particularly in populations with varied gut microbiota compositions due to different dietary or environmental factors. This knowledge could lead to approaches involving microbiota modulation that optimize vaccine responses across different demographic groups.

In summary, the relationship between the gut microbiota and oral vaccines such as rotavirus, cholera, and potentially ETVAX, is a crucial aspect of vaccine development and deployment. It opens up new research avenues for leveraging the gut microbiome to optimize vaccine efficacy.

## 18. Discussion and Conclusions

We described the reasoning for several key trial design features important to The Gambia trial. These features include study location, vaccination age, vaccine dose and number of doses, use of an adjuvant, definition of vaccine-preventable outcomes, and provide an update on the status of the ongoing phase 2b ETVAX^®^ clinical trial. Several future considerations for ETEC trials are also given. We anticipate that this information from the Gambian trial will be useful for the design of future field-based efficacy trials for enteric vaccines.

It has been a little over half a century since ETEC was recognized as a human pathogen and a vaccine against it will certainly be an important step forward in the control of ETEC infections. Ultimately, the control of enteric infections will probably be achieved through combining antigens for multiple pathogens into combination vaccines. Whole cell products such as ETVAX^®^ may facilitate this process by being combined and, where appropriate, engineering expression of conserved homologous and heterologous antigens.

## Figures and Tables

**Table 1:** Clinical Development of ETVAX^®^ for Children.

Study Number	Phase/Location	Age Groups (No)	Dose (Doses)	Key Finding (Reference Number)
OEV-120 (EudraCT ^1^: 2009-015741-23)	Phase I, Gothenburg, Sweden	19–46 years (58)	3.4 × 10^10^ bacteria over expressing CFA/I with 1 mg LCTBA and 4× dose (two)	CFA/I & LCTBA (prototype vaccine) safe and immunogenic [26]
OEV-121 (EudraCT ^1^: 2011-003228-11; CTN91363076)	Phase 1/Gothenburg, Sweden	18–45 years (129)	8 × 10^10^ bacteria, 10 µg or 25 µg dmLT (two)	ETVAX^®^ ±dmLT was safe and well tolerated; dmLT enhanced anti-CF responses, primarily CS6 [22]
OEV-122 (NCT ^2^:02531802)	Phase 1/Mirpur, Bangladesh	18–45 years (45)	Adults dose: 8 × 10^10^ bacteria, 1 mg of LCTBA, 10 μg dmLT (two)	ETVAX^®^ with or without dmLT induced strong systemic and mucosal immune responses [27]
OEV-122 (NCT ^2^:02531802)	Phase 2/Mirpur, Bangladesh	6–11 months (200) 12–23 months (100) 24–59 months (130)	One-half, one-quarter, or one-eighth adult dose with none, 2.5 μg, 5.0 μg, or 10.0 μg dmLT (two)	Safe and immunogenic; dmLT improved immune response in infants [23,28,29]
OEV-123 (NCT ^2^ :03729219)	Benin, West Africa	18–65 (749)	8 × 10^10^ bacteria, 1 mg of LCTBA, 10 μg dmLT (two)	Safe and immunogenic [25]
OEV-124 (PACTR ^3^: 201905764389804)	Lusaka, Zambia	18–45 years (40) 6–9 months (146) 10–24 months (60)	Full dose (adults) with 10 μg dmLT (one), 1/4, and 1/8 adult dose with 2.5 μg (three)	Safe, immunogenic, 3rd dose boosted immunogenicity [24]

^1^ European Union Drug Regulating Authorities Clinical Trials number. ^2^ National Clinical Trial identifier of ClinicalTrials.gov. ^3^ Pan African Clinical Trials Registry number

**Table 2:** Ant-CTB serum IgA titer ^1^ in U.S. Travelers upon arrival in Guatemala or Mexico and risk of moderate to severe ETEC traveler’s diarrhea.

Anti-CTB IgA Titer at Arrival ^1^	Number of Travelers	Moderate-Severe ETEC Diarrhea Cases ^2^	Relative Risk (95% Confidence Intervals)
≤40	279	10	Reference
41–120	31	2	1.80 (0.41, 7.85)
121–360	73	2	0.76 (0.17, 3.41)
361–1080	81	0	0.16 (0.01, 2.75)
>1080	185	1	0.15 (0.02, 1.17)

^1^ Anti-CTB titers in arrival serum regardless of vaccination status. ^2^ Moderate to severe diarrhea = ETEC recovered from subjects as a sole pathogen sharing vaccine antigen during diarrhea episode; ≥3 unformed or liquid stools in a 24 h period and moderate to severe GI symptoms or illness causing a change in normal daily activity. Note: adapted from Walker and Bourgeois, Front Immunol 2023, 14, 1125102 ([76]).

## Data Availability

Data are contained within the article.
